# Mechanism of Zinc Excitotoxicity: A Focus on AMPK

**DOI:** 10.3389/fnins.2020.577958

**Published:** 2020-09-15

**Authors:** Yang-Hee Kim, Jae-Won Eom, Jae-Young Koh

**Affiliations:** ^1^Department of Integrative Bioscience and Biotechnology, Sejong University, Seoul, South Korea; ^2^Neural Injury Research Laboratory, Department of Neurology, University of Ulsan College of Medicine, Seoul, South Korea

**Keywords:** stroke, oxidative stress, apoptosis, lysosome, mitochondria, LKB1

## Abstract

Over the last 20 years, it has been shown that complex signaling cascades are involved in zinc excitotoxicity. Free zinc rapidly induces PKC activation, which causes reactive oxygen species (ROS) production at least in part through NADPH oxidase. It also promotes neuronal nitric oxide synthase, thereby increasing nitric oxide (NO) production. Extracellular signal-regulated kinase activation and Egr-1 transcription factor activity were quickly induced by zinc, too. These concurrent actions of kinases consequently produce oxygen free radical, ROS, and NO, which may cause severe DNA damage. Following the excessive activity of poly(ADP-ribose) polymerase-1 depletes NAD^+^/ATP in the cells. Zinc excitotoxicity exhibits distinct characteristics of apoptosis, too. Activation of caspase-3 is induced by liver kinase B1 (LKB1)-AMP-activated kinase (AMPK)-Bim cascade signaling and induction of p75NTR receptors and p75NTR-associated Death Executor. Thus, zinc excitotoxicity is a mechanism of neuronal cell death showing various cell death patterns. In addition to the above signaling cascades, individual intracellular organelles also play a crucial role in zinc excitotoxicity. Mitochondria and lysosomes function as zinc reservoirs, and as such, are capable of regulating zinc concentration in the cytoplasm. However, when loaded with too much zinc, they may undergo mitochondrial permeability transition pore (mPTP) opening, and lysosomal membrane permeabilization (LMP), both of which are well-established mechanisms of cell death. Since zinc excitotoxicity has been reported to be associated with acute brain injuries, including stroke, trauma, and epilepsy, we performed to find the novel AMPK inhibitors as therapeutic agents for these diseases. Since we thought acute brain injury has complicated neuronal death pathways, we tried to see the neuroprotection against zinc excitotoxicity, calcium-overload excitotoxicity, oxidative damage, and apoptosis. We found that two chemicals showed significant neuroprotection against all cellular neurotoxic models we tested. Finally, we observed the reduction of infarct volume in a rat model of brain injury after middle cerebral artery occlusion (MCAO). In this review, we introduced the AMPK-mediated cell death mechanism and novel strategy for the development of stroke therapeutics. The hope is that this understanding would provide a rationale for acute brain injury and eventually find new therapeutics.

## Introduction

More than 50 years ago, John Olney reported a seminal finding that natural amino acid, monosodium glutamate (MSG) could cause neuronal death in immature murine brains (Olney, 1969a). Following investigations showed that neuronal excitation by glutamate is essential for its neurotoxic effect (Olney, 1969b; Olney and Sharpe, 1969; Burde et al., 1971), and hence the term “excitotoxicity” was coined (Olney, 1969a). A series of studies then demonstrated that specific measures inhibiting excitotoxicity protect against neuronal death in models of acute brain injuries (Rothman and Olney, 1986). Although Olney initially considered the importance of Na influx and energy depletion as the main ionic mechanism for excitotoxicity, subsequent studies demonstrated that excessive calcium influx predominantly via the N-methyl-D-aspartic acid (NMDA) subtype of glutamate receptor mediates most of excitotoxicity at least under brief exposure conditions (Choi, 1987). Interestingly, while glutamate also induces Na influx via both NMDA and α-amino-3-hydroxy-5-methyl-4-isoxazolepropionic acid (AMPA)/kainate receptors, resulting in massive cellular swelling, within a brief period (a few hours), such cellular swelling seems largely reversible (Choi, 1987). Hence, calcium has been considered the primary ionic mediator of excitotoxicity (Choi et al., 1988).

However, a growing body of evidence supports the idea that endogenous zinc plays a role as another ionic mediator of excitotoxic neuronal death (Weiss et al., 1993; Koh et al., 1996). Chelatable zinc is enriched in glutamatergic synaptic vesicles and released with neuronal activity (Assaf and Chung, 1984; Howell et al., 1984; Wenzel et al., 1997). Following the release, some of zinc may enter neurons via calcium-permeable channels such as NMDA channels, voltage-gated calcium channels, or GluR2-lacking AMPA/kainate channels (Sensi and Jeng, 2004). Furthermore, injuries such as oxidative stress release zinc from zinc-binding proteins such as metallothioneins and various organelles (Chung et al., 2007; Hwang et al., 2008). Usually, the approximate concentration of free zinc in the cytoplasm ranges from ten to hundreds of picomoles per liter (Peck and Ray, 1971; Magneson et al., 1987; Simons, 1991; Frederickson et al., 2005; Bozym et al., 2006; Krezel and Maret, 2006; Colvin et al., 2008; Vinkenborg et al., 2009; Qin et al., 2011). Under the stimulation conditions, cellular zinc levels increase and reach 2 nmol/L concentrations (Maret, 2013). Unless the buffering capacity is reduced, cellular zinc levels return to the normal concentrations within minutes (Li and Maret, 2009). However, under the pathological conditions, increased cellular zinc levels are sustained, which induces neuronal toxicity (Sensi et al., 1997, Sensi et al., 2003a; Canzoniero et al., 1999; Aizenman et al., 2000). The relevance of zinc excitotoxicity in acute brain injury was first demonstrated in a rat model of transient global ischemia (Koh et al., 1996). Increases in the level of free zinc are cytotoxic via various signaling cascades (Szabó and Dawson, 1998; Kim et al., 1999; Noh et al., 1999; Park and Koh, 1999; Noh and Koh, 2000; Park et al., 2000; Sheline et al., 2000).

## Roles for Kinases in Zinc Excitotoxicity

**FIGURE 1:**
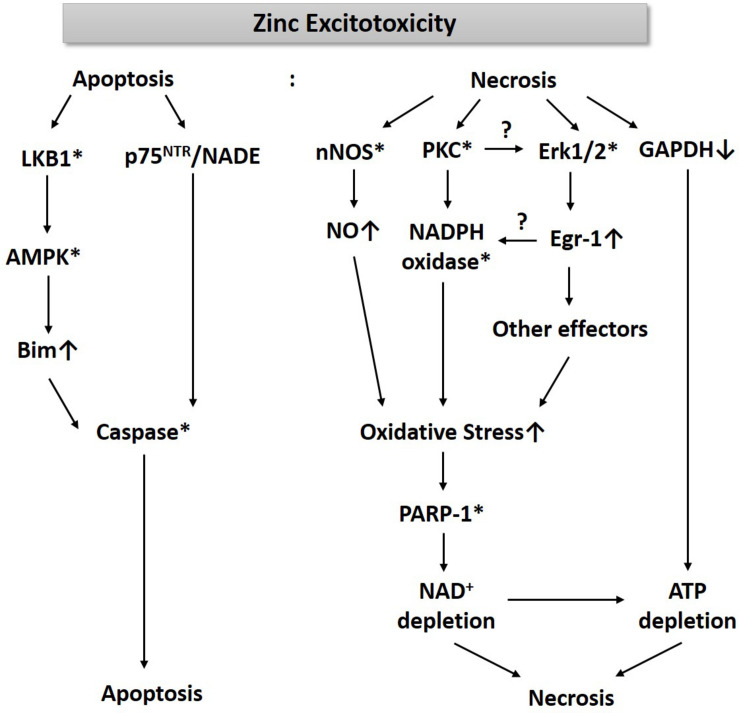
A diagram for the mechanism of zinc excitotoxicity. Zinc excitotoxicity has characteristics of necrosis and apoptosis. Zinc rapidly activates nNOS, PKC, and Erk1/2, which increases NO and ROS (Noh et al., 1999; Park and Koh, 1999; Kim and Koh, 2002). These oxidative stress-induced PARP-1 over-activation and resultant NAD^+^/ATP depletion (Kim and Koh, 2002). Zinc also depletes ATP through the inhibition of GAPDH and glycolysis (Sheline et al., 2000). These events may lead to necrosis. Another pathway of zinc toxicity is apoptosis. Zinc induces LKB1-mediated AMPK activation and then increases Bim expression (Eom et al., 2016). p75NTR and NADE are also induced by zinc (Park et al., 2000). By activating caspases, this pathway induces apoptosis (Kim et al., 1999). * represents activation.

For the past three decades, we have been studying cell death mechanisms caused by exposure to excessive zinc in cultured cortical neurons and glia. These studies have taught us that an increase of free zinc levels in neurons or astrocytes rapidly activates several kinases such as PKC and extracellular signal-regulated kinase (Erk1/2), which appears critical for the resultant cell death (Figure 1). While PKC activation enhances the activity of nicotinic adenine dinucleotide phosphate (NADPH) oxidase (Noh et al., 1999; Noh and Koh, 2000), Erk1/2 induces Egr-1, one of the immediate early zinc finger translation factors (Park and Koh, 1999). Signaling through PKC and Erk1/2 increases the production of oxygen free radicals. Additionally, zinc rapidly increases nNOS expression and activity in neurons, leading to an increase in nitric oxide (NO) (Figure 1). Conversely, Bossy-Wetzel et al. (2004) showed that NO through the formation of peroxynitrite (ONOO^–^) leads to the release of zinc from intracellular stores, which induces mitochondrial permeability transition pore (mPTP) opening, cytochrome C release, reactive oxygen species (ROS) generation, p38MAP kinase-mediated K^+^ efflux, and resultant neuronal apoptosis. The concomitant increase in ROS and NO can cause severe DNA damage, which induces the activity of poly(ADP-ribose) polymerase-1 (PARP-1). During zinc excitotoxicity, excessive activation of PARP-1 continues, and consequently, NAD^+^/ATP levels in cells rapidly decline, resulting in cell death (Kim and Koh, 2002; Figure 1). Sheline et al. (2000) also reported that glyceraldehyde-3-phosphate dehydrogenase (GAPDH), a key enzyme for glycolysis, was inhibited in the zinc excitotoxicity, resulting in a decrease in ATP (Figure 1). Hence, the eventual cell death mechanism by zinc may involve severe energy depletion.

As described above, zinc excitotoxicity causes a decrease in energy in nerve cells, which may activate AMP-activated protein kinase (AMPK) that senses metabolic stress (Carling et al., 2008). AMPK is a hetero-trimeric complex that consists of a catalytic alpha subunit and two regulatory subunits, beta and gamma. Several isomers of each subunit have been reported (alpha 1 and 2; beta 1 and 2; gamma 1, 2, and 3). In addition to the energy reduction, phosphorylation at alpha subunit by two different upstream kinases, liver kinase B1 (LKB1), or calcium/calmodulin-dependent protein kinase kinase beta (CaMKKβ), increased the enzymatic activity of AMPK (Hardie et al., 2012). 5-aminoimidazole-4-carboxamide ribonucleotide (AICAR) and metformin are known as representative chemical activators, and C75 and Compound C are used as inhibitors (Viollet et al., 2010). Since AMPK inhibitors reduce zinc toxicity, AMPK activation may also contribute to zinc excitotoxicity (Eom et al., 2016). However, in the mechanism of zinc excitotoxicity, AMPK activation appears much faster than the time when the AMP level is significantly reduced (Eom et al., 2016). Instead, LKB1, one of the well-known upstream kinases for AMPK (Hardie, 2004; Mihaylova and Shaw, 2011), activates AMPK in zinc excitotoxicity. We have reported that LKB1-activated AMPK can induce caspase-3 activation through increased expression of Bim protein, one of the pro-apoptotic Bcl-2 family members (Eom et al., 2016). Besides, zinc triggers the expression of NGF, p75NTR receptors, p75NTR-associated Cell Death Executor (NADE) in cortical neuron cultures, which also activates caspase-3 (Kokaia et al., 1998; Park et al., 2000; Figure 1). Thus, zinc excitotoxicity shows not only rapid ROS production and necrosis but also induces caspase-3 activation and apoptosis (Kim et al., 1999). Caspase-dependent apoptosis is the most differentiated characteristic of zinc excitotoxicity because calcium-overload glutamate excitotoxicity does not show caspase-3 activation in cortical cultures (Gottron et al., 1997; Park et al., 2000; Lee et al., 2008).

The studies of zinc excitotoxicity mediated by AMPK showed different results than expected. Firstly, we estimated that AMP reduction in cells would induce AMPK activation, but LKB1 caused AMPK activation in a much faster time (Eom et al., 2016). CaMKKβ is known as another upstream kinase of AMPK, also plays an essential role in zinc excitotoxicity, but it is not linked to AMPK. We observed that CaMKKβ inhibitor, STO-609, significantly attenuated zinc-induced cell death, but STO-609 did not change the phosphorylation levels of AMPK (Eom et al., 2016). The next unexpected thing was that AMPK is related to apoptosis, rather than to oxidative damage followed by ROS and PARP-1. Since ATP depletion appeared as the result of PARP-1 over-activation (Kim and Koh, 2002), we initially thought that AMPK is involved in the necrotic pathway. However, contrary to expectation, AMPK plays a crucial role in apoptosis (Kim and Koh, 2002). In ischemic brain injury, it is known that cell damage in the periphery of the infarct is associated with apoptosis rather than in the central region where blood vessels damaged (Sairanen et al., 2006). Thus, AMPK seems to play a role in the margin of brain infarct by expanding the infarct volume in ischemic brain injury.

## Roles of Intracellular Organelles in Zinc Excitotoxicity

Mitochondria is the central organelle for ATP production, where cellular respiration occurs in which electrons are transported through the electron transport chain, and oxygen is reduced to water. However, under diverse pathological conditions, mitochondria become dysfunctional, and excessive ROS is generated, resulting in cell death (Trushina and McMurray, 2007; Polster, 2013). Mitochondria are also organelles that play a critical role in apoptosis via cytochrome C and apoptosis-inducing factor (AIF) release (Vila and Przedborski, 2003; Polster, 2013). Therefore, many studies have focused on mitochondria as the key player in causing cell death during acute brain injury (Polster, 2013).

The prerequisite of zinc excitotoxicity is an increase in intracellular free zinc levels. For this to occur, there are two possible routes; an influx of extracellular zinc into cells and intracellular release of zinc from zinc proteins and zinc-containing organelles (Sensi et al., 2003b, 2009; Varea et al., 2006). Free zinc in synaptic vesicles are released into the synaptic cleft by synaptic activity and then enter the postsynaptic neurons via calcium-permeable AMPA receptor or voltage-gated calcium channels (Assaf and Chung, 1984; Howell et al., 1984; Wenzel et al., 1997; Sensi and Jeng, 2004). Metallothionein-III, a zinc-binding protein critical for regulating zinc concentration in neurons and astrocytes, may serve as a source for zinc release under oxidative stress conditions (Lee and Koh, 2010). Likewise, intracellular organelles, including mitochondria, lysosome, and ER, also contribute to dampen the toxic free zinc levels in the cytosol by taking up and store intracellular free zinc (Sensi et al., 2003a, Sensi et al., 2009; Varea et al., 2006). However, under pathological conditions such as ischemic brain injury or seizure, excessive levels of free zinc may be taken up into the mitochondria or lysosomes, which triggers ROS generation in mitochondria and membrane permeabilization of mitochondria and lysosome, which leads to the cell death (Trushina and McMurray, 2007; Hwang et al., 2008). In addition, subsequent oxidative stress can release free zinc from mitochondria, which also contributes to cell death (Sensi et al., 1999, 2000; Aizenman et al., 2000; Zhang et al., 2004). Hence, zinc-binding proteins such as metallothioneins and zinc-storing organelles such as mitochondria and lysosomes may function as a kind of double-edged sword in zinc excitotoxicity.

Organelles not only serve as zinc reservoir/source but also interact with signaling cascades of kinases that participate in zinc excitotoxicity. For instance, mitochondria are essential for the AMPK activation in hypoxia, too (Emerling et al., 2009). The upstream kinase of AMPK, LKB1, is activated by ROS generated in mitochondria. Unlike AMPK, LKB1 activation is independent of AMP levels. Consistent with the key role of LKB1 in hypoxia-induced AMPK activation, cells deficient in mitochondrial DNA (ρ0 cells) failed to activate AMPK during hypoxia (Emerling et al., 2009). Consistently, studies have demonstrated that mitochondria dysfunction causes AMPK signaling defects in the hypoxic pulmonary vasoconstriction (HPV) model (Evans, 2006; Evans et al., 2006), a representative example of directly linking mitochondria dysfunction and AMPK pathway.

**FIGURE 2:**
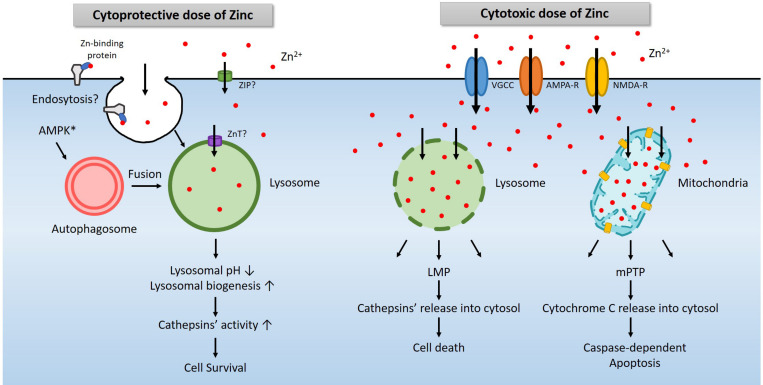
A diagram for the role of lysosomes in zinc-related cell survival and death. Under physiological conditions, a modest increase in cytosolic free zinc translates into a modest increase in lysosomal free zinc due to the function of zinc transporters (Lichten and Cousins, 2009). In addition, endocytosis of zinc-binding proteins also increases lysosomal zinc levels (Rowe and Bobilya, 2000). AMPK contributes to the activation of lysosome via the autophagy pathway (Young et al., 2016; Jang et al., 2018). An increase of free zinc in the lysosome induces lysosomal acidification and activates lysosomal enzymes such as cathepsins. In most cases, these changes promote cell survival (Park et al., 2011; Seo et al., 2015; Lee et al., 2017). However, a high concentration of extracellular zinc enters the cytosol through voltage-gated calcium channel (VGCC), calcium-permeable AMPA receptor (AMPA-R), or NMDA-R, and then lysosomes or mitochondria likely via zinc transporters (Sensi and Jeng, 2004). Excessive zinc in lysosome or mitochondria leads to LMP (Hwang et al., 2008) and mPTP (Wudarczyk et al., 1999; Jiang et al., 2001), which releases cathepsins and other lysosomal enzymes or cytochrome C to causes cell death (Hwang et al., 2008; Mrschtik and Ryan, 2015).

Another possible role of mitochondria in zinc excitotoxicity is to activate the well-established cascade of apoptosis (Calderone et al., 2004). Zinc accumulated in mitochondria can cause mPTP opening (Jiang et al., 2001; Malaiyandi et al., 2005; Gazaryan et al., 2007), resulting in depolarization, swelling, and cytochrome C release and caspase-dependent apoptosis (Jiang et al., 2001). Calcium also induced mPTP opening and cytochrome C release, which was far lower than that induced by zinc (Jiang et al., 2001). That may be the reason we could not observe any pieces of evidence related to apoptosis when we increased intracellular calcium. Thus, through multiple mechanisms, zinc-induced excitotoxicity is affected by mitochondria (Figure 2).

Another essential organelle that plays a crucial role in zinc excitotoxicity is lysosome (Hwang et al., 2008). Free zinc in the cytosol enters not only mitochondria but also lysosomes (Hwang et al., 2008; Sensi et al., 2009). Following exposure to H_2_O_2_ or toxic levels of zinc, the level of zinc in lysosomes rises rapidly and significantly. Afterward, as lysosomal free zinc levels get excessive, the lysosomal membrane becomes more permeable to release proteolytic enzymes into the cytoplasm. Lysosomal enzyme inhibitors were found cytoprotective, supporting the role of lysosomal enzyme activation in cell death under these conditions. Hence the phenomenon called lysosomal membrane permeabilization (LMP) appears to contribute to zinc excitotoxicity (Hwang et al., 2008; Figure 2).

Lysosomes are the actual site for the degradation of cargoes delivered via autophagy, endocytosis, and phagocytosis (Carroll and Dunlop, 2017). Among these, autophagy is regulated by the opposite actions of mammalian target of rapamycin (mTOR) and AMPK (Inoki et al., 2003; Gwinn et al., 2008). Since AMPK is a representative kinase that operates to detect metabolic stress and maintain the energy balance of cells or organisms, activation of AMPK initiates autophagy (Krishan et al., 2015). mTOR signaling is regulated by multiple signals, including growth factors, amino acids, and cellular energy (Hung et al., 2012; Kim and Guan, 2019). mTOR negatively regulates, and AMPK positively regulates the *unc-51*-like kinases 1/2 (ULK1/2) complex. ULK1/2 activates the downstream beclin1 complex, which leads to autophagy induction and then triggers the formation of vesicles called autophagosomes (Ni et al., 2013). These vesicles are fused with lysosomes to degrade the cargoes, including proteins and organelles, to obtain the necessary energy and building blocks in cells (Carroll and Dunlop, 2017). A sub-lethal dose of zinc reduces the pH of lysosomes and promote proteases activity such as cathepsins (Figure 2). Hence, AMPK and zinc may synergistically induce lysosomal function enhancement, which may be beneficial for cell survival under most conditions. However, excessive AMPK activation that may occur in zinc excitotoxicity may further contribute to LMP and cell death. Further studies may be warranted to address this possibility.

## A Role of AMPK in Acute Brain Injury

Although AMPK seems to contribute to zinc excitotoxicity in our experiments (Eom et al., 2016), there is no consensus as to the role of AMPK in various cell death models. *In vitro* neuronal cultures or *in vivo* animal kidney injury models, hypoxia or ischemia/reperfusion injury was reduced by concomitant application of AICAR, a chemical activator of AMPK (Culmsee et al., 2001; Wang et al., 2011; Dugan et al., 2013). Moreover, AMPK is involved in the protective mechanism when melatonin or resveratrol is administered to the ischemia/reperfusion animal model (Wan et al., 2016; Yu et al., 2017). However, many studies have shown that AMPK is involved in triggering toxicity in ischemic brain injury (McCullough et al., 2005; Li et al., 2007; Ronnett et al., 2009). Neuronal death or brain injury is reduced by a chemical inhibitor of AMPK such as compound C or C75, and increased by another AMPK activator, metformin (McCullough et al., 2005; Li et al., 2007, 2010). As discussed above, these discrepant results may occur possibly because cell death mechanisms in these models encompass different mechanisms. Hence, the role of AMPK in a specific condition should be carefully examined.

## A Possible Therapeutic Approach Against Ischemic Stroke With the Focus on AMPK

Since McCullough et al. (2005) found that AMPK plays a role in ischemic brain injury, they proposed C75 and compound C as candidates for stroke treatment (Li et al., 2007). Since we also confirmed that AMPK inhibitors could reduce zinc excitotoxicity, we tried to find noble AMPK inhibitors as therapeutic candidates for ischemic brain injury. Using the virtual screening method, we searched for a chemical library to find chemicals likely to bind to the active sites of AMPK alpha 2. As a result of the screening, 118 chemicals were selected. Subsequently, after selecting 40 inhibitor substances through AMPK enzyme assay, we observed whether these 40 chemicals reduce zinc excitotoxicity comparing with compound C, a well-known chemical inhibitor for AMPK. Seven chemicals significantly inhibited zinc toxicity, but there was no discernable structural similarity (Eom et al., 2019).

Research on the development of a drug for stroke has been actively conducted for the past 30 years. Many research groups tried to develop glutamate antagonists or antioxidants as therapeutic agents (Lalkovicova and Danielisova, 2016). However, all of these clinical trials have failed. The cause of the failure is that ischemic brain injury is not a simple phenomenon caused by a single mechanism. It likely involves various toxic mechanisms, including zinc excitotoxicity, calcium-overload excitotoxicity, ROS-mediated oxidative stress, apoptosis, and LMP. Even if a drug successfully controls a single mechanism, patients may fail to benefit with a meaningful neuroprotective effect since other toxic mechanisms are still active. Therefore, we tried to select chemicals that can suppress various types of neuronal cell death, including zinc toxicity, glutamate excitotoxicity, oxidative stress, and apoptosis to find the chemical candidates for stroke. Therefore, we examined seven chemicals, whether it can attenuate glutamate- or NMDA-induced excitotoxicity, H_2_O_2_-, or Fe^3+^-induced oxidative stress, staurosporine-, or etoposide-induced apoptosis. We finally chose two compounds, 2G11 and 1H10, that exhibited protective effects in all these neurotoxicity paradigms (Eom et al., 2019).

**FIGURE 3:**
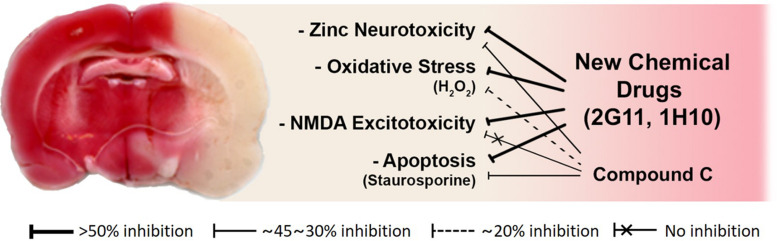
Schematic diagrams of the mode of action of novel candidate neuroprotectants against ischemic brain injury. Both 2G11 and 1H10, but not compound C, the gold-standard AMPK inhibitor, significantly reduced brain damage after middle cerebral artery occlusion in an animal model of stroke (Modified from Eom et al., 2019). Based on the data *in vitro* cortical cultures, the attenuation of zinc excitotoxicity, oxidative stress, or apoptosis by compound C was much lower than that by 2G11 or 1H10. Furthermore, calcium-overload excitotoxicity was not reduced by compound C.

To assess the neuroprotective effects of these chemicals, following focal cerebral ischemia, we used a permanent middle cerebral artery occlusion (MCAO) rat model. We observed that these two chemicals noticeably attenuated ischemic brain injury in the permanent MCAO animal model. Here, we did not see any protective effect of compound C, which may be because the animal model we used experienced quite severe ischemic insults compared with those in other models (Eom et al., 2019; Figure 3). Since based on our results with compound C, the role of AMPK in cortical neuronal cultures was not related to NMDA excitotoxicity. On the other hand, the two lead compounds we selected as above have shown excellent protection in animal models, because they have suppressed not only zinc excitotoxicity, ROS-mediated oxidative stress, and apoptosis but also calcium excitotoxicity (Eom et al., 2019; Figure 3).

Hence, our novel candidates seem to work much better than compound C in a real-world animal model of brain ischemia, likely because they were able to block multiple cascades of cell death. Of note, compared to calcium-overload excitotoxicity or pure apoptosis, zinc excitotoxicity involves more diverse cell death mechanisms (Park and Koh, 1999; Park et al., 2000; Sheline et al., 2000; McLaughlin et al., 2001), and hence may be more useful for neuroprotective drug development as a culture model simulating compound cell death mechanism relevant in acute brain injury.

## Conclusion

We reviewed the role of various kinases and intracellular organelles, including mitochondria and lysosomes in zinc excitotoxicity. In particular, we discussed newly found roles of AMPK in zinc toxicity. Like Zinc, AMPK functions as a double-edged sword in the axis of cell survival-death (Ronnett et al., 2009). In case of chronic neurodegenerative diseases such as Alzheimer’s or Parkinson’s disease, physiological levels of zinc or AMPK activity may promote cell survival through the enhancement of lysosomal function and the resultant reduction of protein aggregates accumulation (Park et al., 2011; Lee et al., 2017; Jang et al., 2018). However, in cases of acute brain injury, excessive zinc influx, and the resultant pathological AMPK activation may trigger cell death (Eom et al., 2016). Thus, in the latter case, alleviating free zinc and inhibiting AMPK may protect against neuronal cell death. Based on these findings, we attempted to discover new AMPK inhibitors as candidate neuroprotective agents in stroke. To find candidates with broad-spectrum efficacy against diverse cell death mechanisms in brain ischemia, we examined the protective effects of chemicals against not only zinc excitotoxicity but also calcium-overload excitotoxicity, oxidative free radical damage, and apoptosis. Two selected compounds showed substantial protective effects in a permanent MCAO model in rats (Eom et al., 2019). The success of our approach may highlight the importance of finding chemicals that can block diverse cell death mechanisms, which are likely involved in acute brain injury such as stroke.

## Author Contributions

Y-HK, J-WE, and J-YK wrote and proofed the manuscript. Y-HK and J-YK conceived of the idea for the manuscript. All authors contributed to the article and approved the submitted version.

## Conflict of Interest

The authors declare that all authors are the inventors of the patent “Pharmaceutical composition for stroke treatment based on AMPK inhibition,” and Y-HK and J-YK participate in Zincure Corp.
